# Promoting self-management, health literacy and social capital to reduce health inequalities in older adults living in urban disadvantaged areas: protocol of the randomised controlled trial AEQUALIS

**DOI:** 10.1186/s12889-018-5219-x

**Published:** 2018-03-13

**Authors:** Laura Coll-Planas, Sergi Blancafort, Xavier Rojano, Marta Roqué, Rosa Monteserín

**Affiliations:** 1 Fundació Salut i Envelliment UAB (Universitat Autònoma de Barcelona), Casa Convalescència. Sant Antoni Maria Claret 171, 4a planta, 08041 Barcelona, Spain; 2 Institute of Biomedical Research (IIB Sant Pau), Barcelona, Spain; 3 Equip d’Atenció Sardenya, EAP Sardenya, Barcelona, Spain

**Keywords:** Social medicine, Public health, Primary care

## Abstract

**Background:**

Older people living in socio-economic deprived urban areas especially suffer the effects of health inequalities but have been insufficiently targeted. Strategies promoted by local primary health care agents might influence health and social behaviours as intermediate social determinants that are modifiable and thus can potentially mitigate health inequalities.

Therefore, we aim to develop and assess the effectiveness of a complex intervention based on a community programme that promotes self-management, health literacy and social capital targeting older people from urban socioeconomically disadvantaged areas in order to improve their self-perceived health as an indicator of health inequality reduction.

**Methods/design:**

Design: A pragmatic multicentre, parallel, randomised controlled trial will be implemented in 16 primary health care centres from six urban areas in neighbourhoods with low-socioeconomic level. Target: community-dwelling aged 60 years or above who perceive their health as fair or poor.

The programme is called “Sentir-nos Bé” (“Feeling well”) and comprises 12 two-hour sessions held once a week in groups of 12–15 people. Group dynamics are designed to promote mutual support, social participation and new knowledge on health literacy and self-management, resulting in meaningful changes in their daily life that positively affect their health and wellbeing.

A sample size of 390 participants, randomised to the intervention or the control group, will be needed to detect a clinically relevant benefit in the primary outcome self-perceived health after 3-month intervention. A follow-up will be conducted at 9 months post-intervention. Participants in the control group will receive usual care and remain in a waiting-list to join the programme once the trial ends. A process evaluation will provide greater confidence in the conclusions about the effectiveness of the intervention. Ethics approval: Clinical Investigation Ethics Committee of the IDIAP Jordi Gol (P15/031). Dissemination: Findings will be disseminated through conference presentations and open-access journals.

**Discussion:**

The project will promote the implementation of evidence-based intervention procedures in future health policy strategies targeting older people while considering the social aspects of inequality.

**Trial registration:**

NCT02733523. Retrospectively registered. Date of registration: April 11, 2016.

## Background

The most disadvantaged socioeconomic groups have generally poorer health status [1]. Cities are the territories that concentrate higher health inequalities and ageing and gender are axes of inequality [2]. Lastly, there is a worldwide demographic shift towards a more aged society and towards urbanization [3]. Therefore, older people living in socio-economic deprived urban areas should be a specific focus for health inequality policies since they especially suffer its effects.

Despite the growing attention on health inequalities since 1980s, and the progress made in the last years in the development of policies and interventions to reduce them, strategies to reduce health inequalities are heterogeneously implemented and the group of older people has been insufficiently targeted [4].

Current evidence is strong in explaining the contribution of specific factors to health inequalities but not on the effectiveness of policies and interventions to reduce them, since it does not often fulfil the highest scientific standards. Consequently, more rigorous evidence-based approaches are needed to inform policymaking in this area [5]. In this respect, the emerging methodologies that address complex interventions could be valuable [6].

It has been raised that focusing on intermediate effects of interventions that target indicators such as the prevalence of health determinants can be useful to reach the WHO goal of reducing health inequalities by 25% [7, 8]. In this vein, self-perceived health could be a useful indicator since it correlates with general health status, mortality and morbidity, as well as health inequalities [9–11]. Likewise, health promotion and salutogenesis emphasize the need to improve the perception of health, along with wellbeing and quality of life [12, 13].

It is acknowledged that the reduction of socioeconomic inequalities in health requires comprehensive strategies with sustained actions addressing social determinants of health instead of isolated actions. Innovative strategies have been identified as effective in five policy areas: policy steering mechanisms, labour market and working conditions, territorial approaches, consumption and health-related behaviour and health care. Hence, two main strategies have been defined: addressing upstream factors with actions related to income, employment and education, and addressing downstream factors with health-related actions targeting health-related behaviours such as food consumption, smoking, and physical exercise. Healthy lifestyles can be promoted by universal and targeted approaches. Targeted approaches can be part of more general strategies and can be directed towards specific groups in terms of age, sex and other socio-demographic characteristics such as living in lower socioeconomic conditions [5].

Promoting **self-care habits** to increase healthy nutrition and physical activity among socioeconomically disadvantaged older people should contribute to reducing their increased risk of morbidity, mortality and disability related with health inequalities [14]. Likewise, adopting measures to improve personal and community health has been linked with the level of knowledge, personal skills and confidence. Accordingly, **health literacy** has been defined as the cognitive and social skills that determine the motivation and ability of individuals to gain access, understand and use information in ways that promote and maintain good health [15]. Low health literacy is related with poor participation in preventive and health promotion activities, worse chronic disease self-management, an increase of hospital stays, and higher mortality and morbidity [16]. Therefore, specific interventions to increase health literacy could contribute to self-care habits.

Accordingly, despite social determinants of health are mainly outside the health care system, health-related actions such as **interventions promoted by local primary health care agents** may be of special interest to influence intermediate determinants of health inequalities that are potentially modifiable such as health-related behaviours, i.e., self-care habits and health literacy [17].

Additionally, **social capital** is considered a social determinant of health, cross-cutting the structural and intermediary determinants, with features linked to both [18–20]. Thus, strengthening social capital has been also included in comprehensive packages of policies and interventions to reduce inequalities in health [21]. Nevertheless, there are controversies on the definition of social capital and its implications on health inequalities. In this study, **social capital** follows Putnam’s approach [22] and is defined according to the conceptual model developed by Islam and adapted to ageing by Nyqvist [23] as an umbrella concept that includes individual (family and friends) and collective (community) social resources, as well as structural (social networks, contacts and participation) and subjective (social support and sense of belonging) aspects. Several observational studies have shown social capital components as consistent protective health factors regarding a variety of outcomes such as mortality and self-perceived health [24, 25]. Whereas the evidence from randomized controlled trials assessing health effects of social capital interventions is still uncertain but promising [26]. Thus, social capital could be part of a multi-component strategy to address health inequalities.

Finally, **social capital components, self-care and health literacy** could reinforce each other reaching synergistic effects on health outcomes, e.g., social support increases self-care while health literacy is associated with healthier habits and higher social participation [26]. Accordingly, after an initial intervention design based on these three pillars, a pilot study was conducted to explore the feasibility of the intervention, the recruitment strategy and evaluation [27].

### Rationale of the study

There is a need to build new intervention strategies based on modifiable intermediate factors to reduce health inequalities. The design of these strategies should consider the synergistic effects of self-care, social capital and health literacy. The effectiveness of these interventions should be robustly assessed considering their complexity to build an evidence-base for public health policy and practice.

Therefore, we aim to **develop and assess the effectiveness of an intervention** designed to promote self-management, health literacy and social capital targeting older people from urban socioeconomically disadvantaged areas who perceived their health as fair or poor in improving their self-perceived health as indicator of reducing health inequalities.

Secondarily, we will assess the effectiveness of the intervention in the improvement of self-management, health literacy and social capital (i.e., social support and social participation), health-related quality of life, loneliness, depressive symptoms and psychological well-being. In addition, the effectiveness of the intervention will be assessed on specific subgroups: women, oldest people, and people with lowest educational and socioeconomic levels. Finally, we aim to identify profiles of participants according to their patterns of health behaviours, health literacy and social capital, considering gender, educational and socioeconomic level and contextual factors, and we will explore whether and how these profiles respond to the intervention.

## Methods and analysis

### Study design and setting

To achieve these objectives, a pragmatic multicentre, parallel, randomised controlled trial (RCT) will be carried out. The study protocol adheres to the SPIRIT recommendations [28]. The trial will be implemented in 16 primary health care centres (PCC) from six urban areas. We have prioritized neighbourhoods with low-socioeconomic levels with established community programmes to reduce health inequalities [29, 30].

### Participants

Participants are eligible if: they are community-dwelling aged 60 years or above and perceive their health as fair or poor. Screening on self-rated health will be performed through the first item of the SF-36 questionnaire: “In general, would you say your health is: (1) Excellent, (2) Very Good, (3) Good, (4) Fair or (5) Poor”.

Participants will be excluded if: they need help to go to the PCC; have cognitive impairment or diagnosed dementia; have a medical condition that contraindicates physical activity; have any severe mental health problem that hinders participation in a group dynamic; have an end-of-life situation.

### Recruitment

PCC of the prioritized neighbourhoods are contacted to offer their involvement in the programme. The research team will inform the whole team about the background, the aims of the study, eligibility criteria and the recruitment strategy.

Professionals from PCC will be selected on a voluntary basis to collaborate as outcome assessors or observers of the intervention. Each PCC is committed to recruit 30 participants. Professionals can identify potential participants in routine visits or actively calling known patients that might fulfil eligibility criteria. As additional recruitment strategies, posters and videos are displayed in the PCC advertising the study. A professional from the PCC will explain the trial to interested patients and screen for the eligibility criteria. Those participants who fulfil the criteria and agree to participate will be given the information sheet about the study and the informed consent.

### Randomisation and blinding

**Fig. 1:**
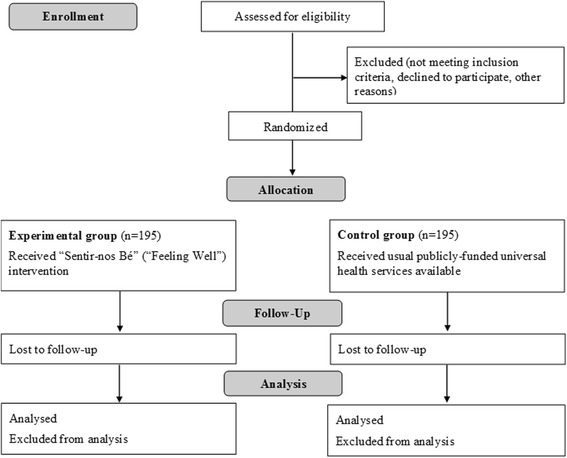
Trial Consort flow diagram

Participants who sign the informed consent will be scheduled for the baseline assessment. Once the participant has been included in the study, assigned an identification code, and has completed the study baseline assessment, the participant will be randomised to one of the two study groups: intervention and control group (fig. 1). Concealed randomisation will be conducted centrally at FSiE-UAB, using a computer-based random-block randomisation scheme, stratified by PCC. In each PCC, one experimental group and one control group will be set up. Participants and professionals conducting the group-based intervention will remain unblinded, likewise participants can easily reveal their group allocation and thus blinding outcome assessors would be hard to sustain.

### Interventions

#### Experimental group

The intervention is a community program with multiple components, thus a complex intervention. A feasibility study was conducted to test the initial intervention design and the intervention was optimized for the full trial accordingly [27]. The final intervention is described in accordance with the TIDieR guidelines below and in the Table 1 [31].

**Table 1:** Sessions, contents, materials and procedures of “Sentir-nos Bé” community programme

Session number	Contents	Materials and procedures
1	Health and self-care	The instructor presents the programme to the participants and they introduce themselves to the group. Using photos of daily activities related to self-care, participants share judgements, views and ideas about self-care. Participants are invited to bring a personal object from home in the next session to introduce themselves. At the end of each session, participants create cooperatively a board with paintings, handcrafts, small writings and thoughts, etc.
2	Physical activity	Participants explain briefly to the group the personal object brought from home. Participants set and share a specific goal related with their daily life to be fulfilled during the week. Using local maps, participants identify and share physical activity assets, discuss about benefits and limitations of doing physical activity and agree on a group walk in the neighbourhood for the next session.
3	First local trip	The group does a round walk starting and ending at the primary care centre visiting interesting places where to do physical activity, e.g., parks or outdoor gyms.
4	Emotional health	Participants share the fulfilment of their personal goal. If they do not succeed, the instructor carries out a problem solving technique (each session until the end of the programme, excepting the local trips). The group discusses and shares strategies to cope with emotions and feelings. A relaxation technique practice is carried out.
5	Healthy eating habits	Participants share their nutritional habits and tips to eat cheaper and healthier. The group shares and discusses about the places in the neighbourhood where they normally buy. Participants agree upon the market or the supermarket to visit in the next session.
6	Second local trip	The group visits a local market or supermarket. During the visit, participants share and discuss about their habits and preferences when buying. The instructor and the observers give advice about nutritional facts and reading labels.
7	Loneliness and social relationships	Using a variety of photos, participants share their thoughts and perceptions about loneliness and social relationships. The group splits into subgroups that are asked to create a story based on key words related with social relationships.
8	Participation in the community	A participant reads out loud the story of “La Lita”, an old woman engaged in the community of a low socio-economic neighbourhood. Using local maps, participants identify and share community assets, discuss about benefits and limitations of social participation and agree upon a group visit to a local community asset for the next session.
9	Third local trip	The group visits a community asset. A volunteer from the centre explains to the group the activities carried out. Participants are invited to engage in social activities.
10	Personal autonomy	The group shares their thoughts and beliefs about personal autonomy discussing practical cases presented by the instructor.
11	Communication with health professionals	The group watches four short videos that explain how to prepare the medical encounter. Participants share and discuss their thoughts and feelings when visiting a health professional, as well as their personal knowledge about health and social resources.
12	Group discussion	The group shares and discusses their thoughts and feelings about the programme. Participants are invited to make an imaginary gift to someone else from the group. The group shares a farewell meal.

**Fig. 2:**
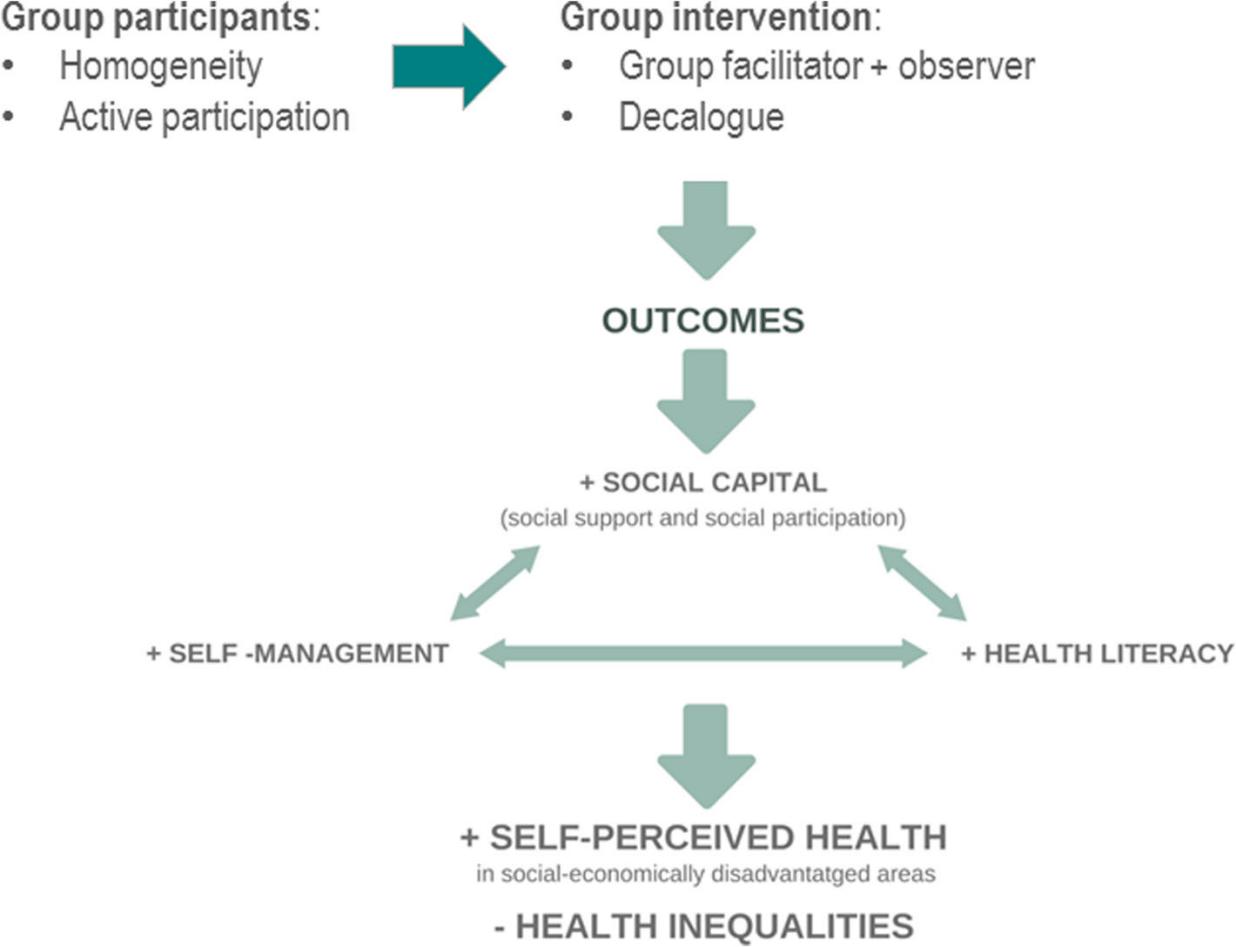
Conceptual framework of the intervention

The programme is called “Sentir-nos Bé” (“Feeling well”) and it is aimed at promoting social support and participation (i.e., social capital), self-management and health literacy as intermediate factors between social determinants and health outcomes with potential to mitigate health inequalities. The conceptual framework of the intervention is described in fig. 2.

The intervention methodology will be participatory with the purpose to promote the empowerment among participants. Group dynamics are designed to promote mutual knowledge and support, trust among participants, exchange of personal experiences among peers and the acquisition of new knowledge related to health literacy and self-management in order to lead to behavioural changes in their daily life that are meaningful for them and that positively affect their health and wellbeing. Health and social care professionals (five nurses, two social workers and two general practitioners) with experience working in the PCC will be previously trained as group facilitators by the research team during a two-day workshop. The training is based on how to apply the intervention guide.

The intervention consists in 12 sessions that are held weekly for 2 h and facilitated in groups of 12 to-15 people. The programme will be delivered in publicly funded PCC and in different locations of the neighbourhood. Specifically, nine of the 12 sessions will be delivered in the centre, and three sessions consist on local trips and are delivered in: a public space where to practice physical activity (park or pedestrian paths), a supermarket or a market and a community asset that offers social activities that can be of interest to the participants.

The intervention guide sets up a frame for the facilitators to conduct the group dynamics following common principles specified in a decalogue and an homogenous structure, while considering participants’ experiences, values and expectations. For example, several activities conducted as a group are highly personalized such as goal setting to change a daily habit to feel better, bringing personal belongings to introduce themselves and making an imaginary gift to another participant. Within the participatory approach, the locations of the three local trips are chosen and agreed by the group based on previous sign posting and mapping of local community assets, thus allowing a tailoring to specific interests and preferences of each group, as well as to the local context.

One or two health or social care professionals from the PCC will be observers of the intervention. They will complete an observation log including quantitative and qualitative measures of implementation such as fidelity and adherence.

### Control group

During the study, the participants from the waiting-list control group will receive the usual publicly funded universal healthcare services available to all population. At the end of the study, these participants will be offered the “Sentir-nos Bé” programme as a compensation for their time spent being assessed.

### Outcomes

Assessments will be conducted by health or social care professionals from the PCC who are not involved with the intervention delivery or observation. All outcome assessors will receive training in interview skills and outcome measure administration by the PI prior to the start. Outcomes will be measured at the following time points: baseline (T_0_), after the intervention is completed (T_1_) and 9 months after the end of the intervention (T_2_).

### Primary outcome

The primary outcome of the study is self-perceived health and will be estimated through the first item of the SF-36 questionnaire considering good self-rated health if they answer (1) Excellent, (2) Very Good or (3) Good, and bad self-rated health if they answer (4) Fair or (5) Poor.

For an overview of primary and secondary outcomes, outcomes measures, instruments and assessments time points, see Table 2.

**Table 2:** Overview of outcomes, outcome measures, instruments and assessments time points

Outcomes	Outcome (measures)	Instrument	Assessment time points
Personal information	Age, gender, living arrangement, civil status, place of birth, educational background, job, medical conditions	Primary care records Self-report	T_0_
	Changes in living arrangement and civil status		T_1_, T_2_
Primary outcomes	Health status	SF-12 EQ-5D visual analog scale	T_0_, T_1_, T_2_
Secondary outcomes	Quality of life	SF-12	T_0_, T_1_, T_2_
	Social capital	European Values Survey	T_0_, T_1_, T_2_
	Social support	Inventory of social resources in the elderly	T_0_, T_1_, T_2_
	Social participation	Loneliness Este II Scale-Subjective Social Participation Index	T_0_, T_1_, T_2_
	Care and self-care	Self-care semFYC survey Appraisal of Self-Care Agency Scale	T_0_, T_1_, T_2_
	Emotional well-being	Warwick-Edinburgh Mental Wellbeing Scale (WEMWBS)	T_0_, T_1_, T_2_
	Loneliness	Gierveld and De Jong scale Social support perception scale	T_0_, T_1_, T_2_
	Depression	Geriatric depression scale (GDS-5)	T_0_, T_1_, T_2_
	Physical activity	The International Physical Activity Questionnaire (IPAQ)	T_0_, T_1_, T_2_
	Health literacy	Health literacy scale (HLS-EU-16)	T_0_, T_1_, T_2_
	Medication consumption Use of social and health resources	Primary care records Self-report	T_0_, T_1_, T_2_

### Sample size

A sample size of 390 participants, randomised to the intervention or the control group of 195 participants, will be needed to detect a clinically relevant benefit in self-perceived health after 3-month intervention. Self-perceived health is dichotomized as good health (including excellent, very good and good) and poor health (including fair and poor). The clinically relevant change has been defined as a 10% increase in the prevalence of participants who consider their health as good. The sample size has been estimated on a statistical power of 80%, a significance level of 95%, and an estimated dropout rate of 20%. A prevalence of 20% of bad self-perceived health in the ESCA 2012 in socio-economic class IVa was considered [32]. The calculations were performed with the “ene 3.0” programme.

#### Data collection

Data collected during the study period will include a questionnaire completed by a face-to-face interview with a researcher at baseline, post-intervention and follow-up points. The time for completion will be documented. Participants will be contacted by telephone prior to each follow-up point, to arrange a convenient time for completion with the researcher. All patient data will be kept in strict confidence and managed in accordance with the Spanish Data Protection Organic Law 1999. The data will be stored for 5 years after the end of the study. Following completion of the trial, a cleaned anonymised data set will be shared on request. A complete schedule of enrolment, interventions and assessments times is provided on Table 3.

**Table 3:**
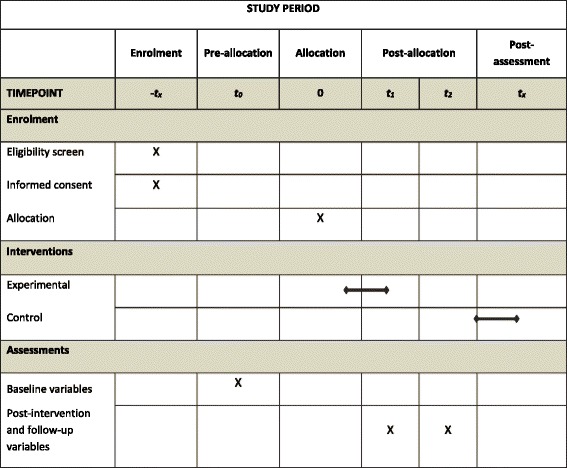
Schedule of enrolment, interventions, and assessments

#### Data analysis

Reporting of the results will follow the CONSORT guidelines for randomised controlled trials [33].

First of all, a descriptive analysis will be conducted to characterize the intervention and the control group regarding socio-demographic, health and psychosocial variables.

The efficacy analysis will assess changes in the primary and secondary outcomes between the baseline and 3 months assessments comparing study arms (experimental and control group) with mixed linear models with repeated measures. Linear models will be used to adjust for covariables of interest, specially to correct differences between arms in the baseline characteristics, if applicable.

A diagonal matrix design will be considered for the between-participants covariance matrices (covariance structure for random factors). The between-participants correlations induced by the group delivery of intervention will be taken into account in the models.

Further testing of the study hypotheses will be conducted through covariance analyses, including in the model relevant covariates (age, gender, educational and socioeconomic levels), and effect modifiers. Estimates of effect will be computed for planned subgroups on age, gender, educational and socioeconomic levels and will be interpreted with caution as exploratory rather than definitive results.

It will follow the intention-to-treat principle, thus including all randomized participants with a baseline assessment regardless of their permanence in the study or their loss to follow-up, withdrawal or drop-out. Furthermore, sensitivity analyses will be conducted. In the first place, a ‘per protocol’ analysis will comprise all participants with no protocol violations and complete data in the measurements of the primary variables at baseline, end of treatment and follow-up. In a second sensitivity analysis, primary variables with a substantial number of missing values (higher than 5%) will be imputed.

All statistical analyses will use two-sided tests of statistical significance; estimates of the size of treatment effects will be presented with confidence intervals and significance tests. Significance levels will be set to a 5% level, applying the Bonferroni correction to adjust for type I error criteria. All analyses will be performed with SPSS version 20.

### Process evaluation

AEQUALIS follows the guidance on “Process evaluation of complex interventions” from the Medical Research Council (MRC) for conducting the process evaluation of the study [34].

The process evaluation of the full trial is aimed at providing greater confidence in conclusions about effectiveness of the intervention by assessing: (a) implementation, i.e., the quantity and quality of what was delivered regarding the community programme; (b) the role of context to understand the generalizability of the results (c) the mechanisms of impact.

Methodologically, quantitative measures of implementation such as fidelity and adherence will be combined with qualitative data focused on implementation challenges, mechanisms of impact and context. Qualitative procedures will comprise: participant observation of a variety of sessions, focus groups with the participants of all intervention groups at the end of the programme, interviews with a purposeful sample of participants with different profiles and follow-up and closing meetings with each team of professionals in charge of facilitate and observe the sessions. Interviews and focus groups will be audio recorded and transcribed. The Framework Method will be applied to conduct a content analysis of the qualitative data [35].

#### Ethics and dissemination

This study has been approved by the Clinical Investigation Ethics Committee of the IDIAP Jordi Gol (P15/031). The Committee will be notified of all substantial modifications to the protocol. The study protocol is registered at ClinicalTrials.gov with the reference number: NCT02733523. Participation is voluntary and all participants will be asked to sign informed consent before the start of the study. At the end of the study, control group participants will be offered the intervention.

One of the tangible products of the study is the intervention guide that facilitates the replication and design of similar interventions, and it can be adapted to different contexts. Each centre can use the data subset from its own centre in order to make secondary analysis. Once the main outcomes of the study are published, data access will be open to other researchers who may be interested.

The AEQUALIS study considers dimensions of participation, public involvement, ethics, gender equality, scientific education and open access included in the framework of Responsible Research and Innovation [36]. The research team will work in collaboration with an advisory committee integrated by three older women with expertise in primary care, adult education and ageing. This committee will be involved in the development of the intervention guide as well as the participant observation of its implementation. As an inequality axis, gender will be considered through all the implementation steps of the research. Although the intervention will be implemented to secure the participation of men -as they are frequently less involved in health promotion and group activities-, a major participation of women will help us to respond to the challenge of the high prevalence of older women living alone -often widows- with a poor self-perceived health and feelings of loneliness and having few opportunities to establish support ties and social networks. Data analysis will be done with men and women subgroups in order to identify possible gender differences in impacts. The research team will contact secondary education schools with the aim of making project disclosure, sensitize about health inequalities, facilitate intergenerational relationships and promote scientific education on ageing research. All publications of the project will be open access.

## Discussion

This study provides a protocol for a multicentre pragmatic randomized controlled trial to assess the effectiveness of an intervention designed to promote self-management, health literacy and social capital in order to improve self-perceived health in older adults living in urban disadvantaged areas.

### Strengths and limitations of this study

To the authors' knowledge, this is one of the few randomized clinical trials conducted in European cities to tackle health inequalities under a community approach [37]. It is based on a complex intervention focused on three pillars (health literacy, self-management and social capital) as potential modifiers of the influence of social determinants on health. It will test its effectiveness on increasing self-rated health in a pragmatic trial, i.e., close to the everyday practice.

Participants and professionals conducting the group-based intervention remain unblinded, likewise blinding outcome assessors is hard to sustain due to the type of intervention. The intervention protocol is standardised in detail, including how to tailor the activities and make them participatory. In addition, the study includes a rigorous facilitator training and mentoring, which enhance the internal validity of the AEQUALIS trial.

This trial includes a process evaluation framed within the MRC guidance to assess how implementation, context and mechanisms of impact affect the outcomes. Accordingly, findings are likely to inform specific health care practices to reduce intermediate factors towards health inequalities, and thus results might be relevant clinically and from a public health perspective and useful for primary health care.

The study will provide evidence on how to implement an intervention tailored to needs and local contexts of older people living in vulnerable situations. The high capacity of tailoring the intervention to the participants and to the local context should allow the programme to be transferred to other contexts. Furthermore, qualitative methodology will help to explore how individual health and social trajectories are linked with inequalities and will provide a better understanding of values, attitudes and personal expectations on self-management, as well as health behaviours of older people in a context of social vulnerability.

The project will promote the implementation of evidence-based intervention procedures in future health policy strategies targeting older people while considering the social aspects of inequality. Indeed, at regional level, the project is aligned with the aim set out in the Catalan Health Plan 2016–2020 on reducing social inequalities measured as an increase of self-perceived health [38]. The results will provide an evidence base to set recommendations addressed to the older population, primary health care professionals and policy makers.

Finally, this project can be used as a model for its biopsychosocial perspective of health with an emphasis on salutogenesis, as a source of wellbeing and good health.

### Trial status

The initial phases of this project started in March 2015. A feasibility study was performed from September 2015 until December 2015. The recruitment for the trial started in January 2016. At the time of submission of this protocol all participants have been included in the study. To date, none of the participants have completed the outcomes assessment.
